# X-ray radiation shielding and microscopic studies of flexible and moldable bandage by *in situ* synthesized cerium oxide nanoparticles/MWCNTS nanocomposite for healthcare applications†

**DOI:** 10.1039/d3ra00067b

**Published:** 2023-03-15

**Authors:** Sarika Verma, Manish Dhangar, Harsh Bajpai, Kamna Chaturvedi, Ranjan K. Mohapatra, Mohd. Akram Khan, Mohammad Azam, Saud I. Al-Resayes, Avanish Kumar Srivastava

**Affiliations:** a Council of Scientific and Industrial Research- Advanced Materials and Processes Research Institute Hoshangabad Road Bhopal M. P. 462026 India drsarikaverma20@gmail.com; b AcSIR-Advanced Materials and Processes Research Institute (AMPRI) Hoshangabad Road Bhopal M. P. 462026 India; c Department of Chemistry, Government College of Engineering Keonjhar 758002 Odisha India rkmohapatra@gcekjr.ac.in; d Department of Chemistry, College of Science, King Saud University PO BOX 2455 Riyadh 11451 Saudi Arabia azam_res@yahoo.com

## Abstract

This research reports a robust method for developing advanced flexible and moldable X-ray shielding bandages by harnessing an *in situ* synthesized polygonal cerium oxide nanoparticles/MWCNTs nanocomposite. The developed advanced hybrid nanocomposite was thoroughly blended with silicone rubber, namely polydimethylsiloxane (PDMS) to form an advanced hybrid gel which was then coated on a conventional cotton bandage to develop an advanced flexible, moldable X-ray shielding bandage. The combined effects were analyzed to determine their unique X-ray reduction properties and were very effective. The linear attenuation value of the developed bandage (untreated cotton bandage coated with CeO_2_/MWCNT/PDMS), varied from 1.274 m^−1^ to 0.549 m^−1^ and the mass attenuation values from 0.823 m^2^ kg^−1^ to 0.354 m^2^ kg^−1^ for kVp 40 to 100 respectively. The improved features of high density and efficiency of protection are because of the binary protective effect of CeO_2_ nanoparticles and MWCNT. The morphological features of the developed material were characterized using various techniques such as TEM, SEM, XRD, and EDXA. The developed bandage is an entirely lead-free product, thin and light, has high shielding performance, flexibility, durability, good mechanical strength, doesn't crack easily (no crack), and can be washed in water. It may therefore be useful in various fields, including diagnostic radiology, cardiology, urology, and neurology treatments, attenuating emergency radiation leakages in CT scanner rooms or *via* medical equipment, and safeguarding complex shielding machinery in public areas.

## Abstract 2

This research reports a robust method for developing advanced flexible and moldable X-ray shielding bandages by harnessing an *in situ* synthesized polygonal cerium oxide nanoparticles/MWCNTs nanocomposite.

## Graphical abstract

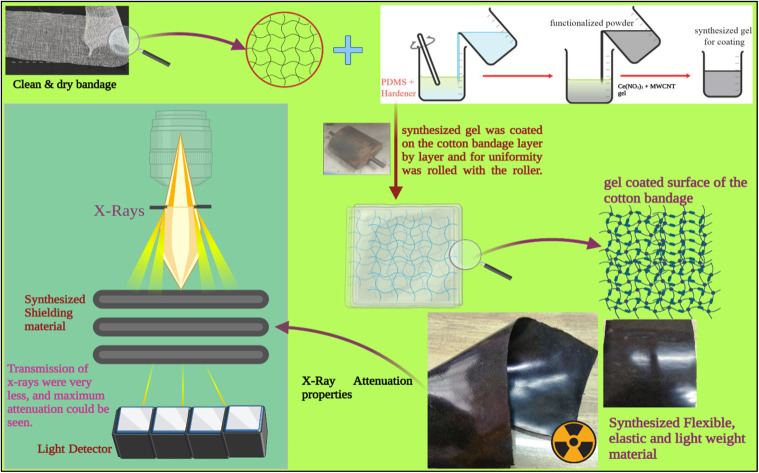

## 1 Introduction

When X-rays pass through the sample material, they are reflected, scattered, and transmitted. Such materials have unique importance in medical fields, especially diagnostic and therapeutic medicine.^1^ These diagnostic applications include CT scan imaging, diagnostic radiology, fluoroscopy, microscopy, *etc.* However, owing to their ionizing properties and incorrect application, these X-rays may have seriously deleterious consequences on the end-users.^1^ They can even induce tumor growing cells, alter DNA, and thus have severe gene mutation effects.^1^ One can minimize these adverse radiation effects by using an appropriate radiation shielding material to attenuate the X-ray radiation energy during their use.

Although non-flexible radiation shielding materials such as tiles, paver blocks, concrete blocks, sheets, and so on are widely accessible, flexible shielding materials such as bandages, scarves, and aprons are few. The non-flexible radiation shielding materials have disadvantages as they are not flexible due to their rigidness and thus have minimal usage. The flexible and mouldable radiation shielding materials are convenient to prepare and handle, therefore play a significant role as effective radiation shielding materials in various sectors like X-rays, CT scanner rooms, defense, electrical and electronic equipment, nuclear research, aerospace, astronomy, industries, hospitals, chemical industries, electricity power plants, *etc.* Further, conventionally, the lead (Pb) based radiation shielding aprons have been utilized as protection equipment by patients, doctors, and operators. The conventional X-ray shielding aprons are made up of lead infused in rubber or vinyl components and a maximum Pb_eq_ of 1 mm. It made them very heavy (>11 pounds), uncomfortable, and challenging to handle, primarily when used for a very long duration. These lead-based aprons are highly toxic, a known carcinogen, and are less durable as they bend easily and thus undergo cracks. The advancements focus on the fabrication of the radiation protection apron decreases the physical stress on the wearer. As a result, scientists are working on non-toxic, lightweight, lead-free aprons comprised mostly of chemical compounds comprising bismuth, tungsten, cerium, barium, tin, antimony, and other elements.^2–5^

In recent years, multi-walled carbon nanotubes (MWCNT) have sparked interest in X-ray shielding applications.^6^ Multiple layers improve the interaction of X-ray radiation with MWCNT, leading to a higher scattering of the radiation and faster energy attenuation.^7^ Moreover, the MWCNT speeds up the dispersion of X-rays due to its reduced weight. Furthermore, the impregnation of heavy metals such as zinc, cerium, bismuth, americium, gadolinium, iron, thallium, or their salts in MWCNTs can improve the shielding effectiveness of these hybrid nanostructures.^8,9^ The presence of many free electrons in the outer shells of these metals increases the likelihood of X-ray photons being scattered *via* photoelectron and compton scattering from the shielding materials' surface.^5,10^

Over the years, the significance of cerium oxide nanoparticles for providing radiation-protection effects during X-ray irradiation *via* gene expression modification and the optical properties of gamma irradiated CeO_2_ nanoparticles solution has been reported.^11,12^ Recently, cerium-oxide-based CNT compounds have been reported to exhibit synergistic protection against X-ray radiation in diagnostic programs.^13^ Huang *et al.* (2018) developed oxidized multiwall carbon nanotube/silicone foam compounds with effective protection against electromagnetic interference and high gamma radiation stability.^14^ Tamore *et al.* (2019) claimed that functionalized multi-walled carbon nanotubes impact the physicomechanical properties of silicone rubber nanocomposites.^15^

Polydimethylsiloxane (PDMS), a high-molecular-weight, non-toxic, non-flammable, inexpensive, transparent, effective, and essential silicone-organic rubber polymer with hydrophobic and hydrocarbon methyl groups, has been utilized to make radiation shielding material.^16,17^ The PDMS possesses excellent chemical and biocompatibility properties and shows good dispersibility in developed material with vacillating substances. As a result, it contributes to developing improved X-ray shielding materials for key applications. Verma and coworkers used PDMS to form a hybrid organic–inorganic gel, which produces a flexible and mouldable X-ray shielding material with the required dimensions.^18–20^

Therefore, the primary objective is to develop advanced flexible and moldable bandages by harnessing *in situ* synthesized cerium oxide nanoparticles/MWCNTs hybrid nanocomposites for healthcare applications.^4^ A chemical *in situ* approach for producing cerium oxide and MWCNT-based nanocomposite was devised. An enhanced X-ray radiation shielding bandage was made by coating the developed hybrid nanocomposite over a conventional cotton bandage. The morphology of the developed bandage was studied by using the SEM and TEM respectively. X-ray radiation shielding attenuation properties were studied for conventional and developed advanced hybrid bandages. Various parameters like half value thickness (HVL), linear attenuation, and mass attenuation were studied and compared with lead-based composites at different kVp (peak voltage).

## 2 Materials and methods

### 2.1. Materials

MWCNT (>95% purity, 20–25 μm length) has been obtained by Nano solution Co., Korea. The chemicals cerium(iii) nitrate hexahydrate, hexamethylenetetramine (C_6_H_12_N_4;_ HMTA) were obtained from Rankem, while cytosine, and sodium hydroxide (NaOH), nitric acid (HNO_3_), sulfuric acid (H_2_SO_4_) is produced locally from industries such as Sigma-Aldrich, Himedia, and Merck respectively. PDMS (Sylgard 184, Dow Corning Corporation) was obtained from Kevin Electrochem, Mumbai, India. The conventional bandage was obtained from the local market of Bhopal, India. All these reagents were utilized as acquired.

### 2.2. Synthesis

To functionalize MWCNTs, a previously published synthesis process was used.^21^ Briefly, for the functionalization process, 5 g MWCNTs were refluxed at 90 °C in a solution containing 150 mL of 3 M HNO_3_ and 3 M H_2_SO_4_ acid in the round bottom flask for 8 h at 600 rpm for harboring oxygenated functional groups, thereby eliminating the amorphous carbon derivatives and other impurities. The contents were filtered, and the residue was rinsed several times in double-distilled water (DDW), then dried for 10 h at 65 °C.

### 2.3. Synthesis of hybrid CeO_2_/MWCNTs nanocomposite

36 g of Ce(NO_3_)_3_·5H_2_O was infused in water to make a clear solution. In addition, 5 g of F-MWCNT was added to the solution mentioned above and left in a 4 h bath ultrasonicator (product code: mub-33; metrex scientific instruments Pvt. Ltd.). Then, 3.5 M HMTA aqueous solution (pH = 9.5), was added drop-wise to the above mixture at 80 °C, under continuous shaking on a magnetic stirrer at 600 rpm. Additionally, 30 mL of aqueous cytosine solution was mixed. This is followed by adding 10 M NaOH solution dropwise and kept for refluxing. Finally, the aforesaid combination was irradiated for 15 minutes in a modified microwave oven reactor set to 300 W. The material was then filtered, and the residue obtained was dried and further annealed at 130 °C in the air for 2 h to obtain dry black-colored powder called advanced hybrid CeO_2_/MWCNTs nanocomposite (Fig. S1†).

### 2.4. Preparation of advanced hybrid gel

To prepare an advanced hybrid gel, the polydimethylsiloxane pre-polymer (PDMS) and 12% hardener (containing SiH radical), *i.e.* silicone rubber, was mixed well. It was then thoroughly blended with functionalized MWCNT and hybrid powder enhanced above (wt%) separately to form a variety of advanced hybrid gels. The formulation was then thoroughly blended. The air bubbles were removed using a pump and desiccators to obtain an advanced homogeneous hybrid gel called PMWCNT 1 and Ce PDRM 2 to prepare advanced flexible and moldable radiation shielding bandages.

### 2.5. Preparation of advanced flexible and moldable radiation shielding bandage

**Fig. 1:**
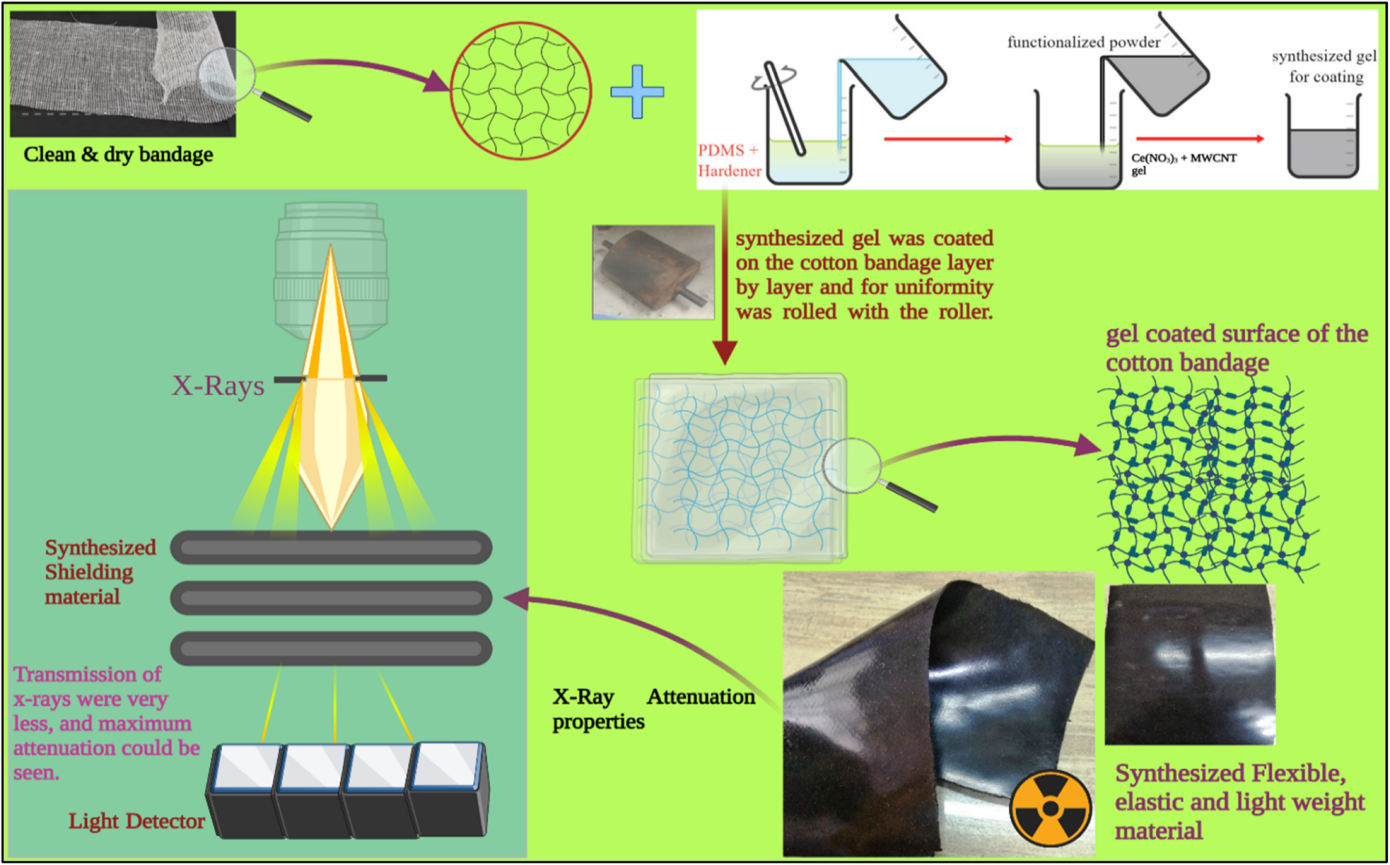
Advanced flexible and moldable radiation shielding bandage with advanced hybrid material, *i.e.*, CeO_2_/MWCNT/PDMS and the conventional bandage coated with gel PMWCNT 1 for developing a bandage with MWCNT/PDMS.

A standard conventional cotton bandage 20 cm long and 11.5 cm wide was thoroughly washed using clear water and dried in the sun. Then, it was mechanically rubbed on both sides with a fine metal brush. The prepared advanced hybrid gels were then applied by hand to the fabric using a paintbrush. A standard conventional cotton bandage was then pressed with a heavy hot metal roller (length: 17 cm; width: 12 cm) for adhering the developed advanced hybrid gel to the cotton bandage. It was then folded and pressed repeatedly three times and dried in the air. This led to the formation of advanced flexible and moldable radiation shielding bandages with advanced hybrid material, *i.e.*, CeO_2_/MWCNT/PDMS (length: 21.3 cm, width: 11.3 cm, and thickness: 2.0 mm) as depicted in Fig. 1. Similarly, the conventional bandage coated with gel PMWCNT 1 for developing a bandage with MWCNT/PDMS was also prepared. The developed bandages were further used for studying X-ray radiation shielding and microscopic properties useful for healthcare applications.

## 3 Characterizations

### 3.1. X-ray shielding attenuation

Using a Nomex Multimeter from PTW, the Dosimetry Company, the created bandages were tested for their X-ray radiation shielding attenuation capabilities. The measurements were made using X-ray photons with energy ranging from 40 to 100 kVp. The X-ray equipment utilized was a Wipro GE DX 525–500 mA, 125 kVp X-ray machine.

The shielding property of the material concerning the x-rays can be calculated through an X-ray machine and dosimeter. Using the absorbance, the transmittance % can be calculated by the formula^22^ -

1 *I*/*I*_0_ = exp^(−*μx*)^

Linear attenuation is a constant that describes the fragment of attenuated incident light in a mono-energetic beam of a material^23^ per unit thickness.

2 μ = [ln(*I*/*I*_0_)]/*x*

where *I*: intensity of X-ray photons that have passed through the shielding material, *I*_0_: transmittance, *μ*: linear attenuation coefficient, and *X*: shielding material thickness (mm).

Mass attenuation quantifies the probability of the interaction between incident light and the matter of the unit mass per unit area.^24–26^

3 *μ*_m_ = *μ*/*ρ*

where, *μ*_m_: mass attenuation coefficient, *μ*: linear attenuation coefficient, and *ρ*: mass density.

A half-value layer is the cross-section of a material required to reduce the specified mass of air of an X-ray or gamma-ray to half of its original value.^23,27^

4 HVL = (0.693/*μ*)

### 3.2. X-ray diffraction-based studies

The components included in functionalized MWCNT, cerium nitrate and the developed advanced hybrid CeO_2_/MWCNT nanocomposite was identified using XRD tests. This was done with CuKα radiation from the D8 advanced X-ray diffractometer throughout the angular range of 5–70 °C.

### 3.3. Transmission electron microscopy

A standard procedure was used to make TEM on a developed advanced hybrid CeO_2_-MWCNT nanocomposite powder sample. At room temperature, 4 mL of water was piped gently into a copper grid tied with a mold and evaporated. Samples were tested using an FEI Tecnai G2 sprit twin transmission electron microscope mounted on a Gatan digital CCD camera (Netherlands) mounted at 80 kv.

### 3.4. Scanning electron microscopy

The three bandages, namely conventional cotton bandage, conventional bandage with MWCNT/PDMS, and conventional bandage coated with advanced hybrid material, *i.e.*, CeO_2_/MWCNT/PDMS were inspected using a JEOL model JEM-35-CF scanning electron microscope. These samples were initially placed on aluminum stubs using carbon tape. Afterward, they were stored to be coated using a tiny layer of platinum to avoid charging before the microphotographs were taken.

### 3.5. EDS and elemental mapping

X-ray scattering analysis of EDX or EDA is sometimes referred to as X-ray energy dispersive X-ray microanalysis EDXMA. This technique is based upon the interaction of an X-ray beam with a developed material wherein it is converted into a single-compliant material in a chemical environment and thus utilized for character specification. The effectiveness of the processes is linked to the fundamental premise that each element has a unique atomic structure that allows for an astonishing collection of peaks in its electromagnetic output spectrum. The conventional cotton bandage, bandage coated with MWCNT/PDMS, and conventional bandage coated with advanced hybrid material, *i.e.*, CeO_2_-MWCNT/PDMS, were examined for EDS and elemental mapping.

## 4 Results and discussion

### 4.1. Density of material

The density of the conventional cotton bandage, conventional bandage coated with MWCNT/PDMS, and conventional bandage coated with synthesized hybrid material, *i.e.*, CeO_2_-MWCNT/PDMS was studied by the standard method.

**Fig. 2:**
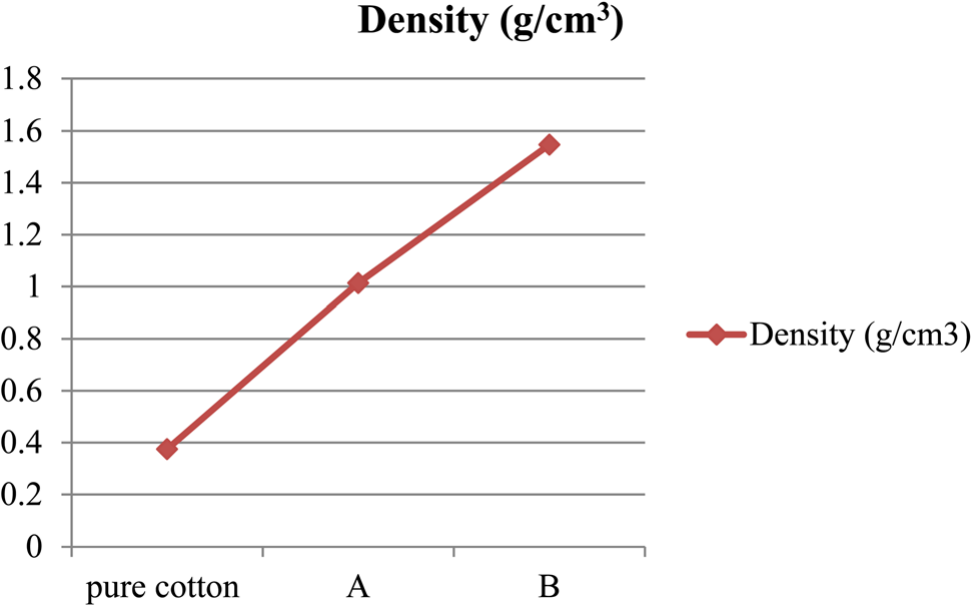
The density of conventional cotton bandage, conventional bandage with MWCNT/PDMS (A), and conventional bandage coated with advanced hybrid material, *i.e.*, CeO_2_-MWCNT/PDMS (B).

The density of different materials was compared and shown in Fig. 2 below. It showed an increase in density when the pure cotton bandage was coated with the shielding material.

The density of the pure cotton bandage was found to be 0.375 g cm^−3^, wherein the density of bandage A was 1.014 g cm^−3^, and the density of pure cotton bandage coated with hybrid material, *i.e.*, CeO_2_-MWCNT/PDMS, was found to be 1.547 g cm^−3^. It showed that the density increases with the use of hybrid materials. Also, it was observed that an increase in density is directly proportional to the percentage attenuation; thus, with the increase in density, the shielding material will attenuate more.^28^ The density of the bandage increases as we increase the layering of the bandage with the hybrid material. Accordingly, the increase in the use of hybrid material fills the interstitial spaces, thereby leading to more compactness and densification of the bandage formed during the coating process.^29^ Therefore, it can be concluded that with an increase in the thickness of the material, there would be an increment in the density and thus leading to more attenuation characteristics in the developed material. Although, there can be a reduction in the flexibility aspects of the developed shielding bandage if more coated material and layers are used. Thus, depending on the end user's requirements, the manufacturers can produce the shielding bandage with the desired characteristics. The newly developed bandage has excellent shielding efficacy, flexibility, durability, and high mechanical strength.^30–32^

### 4.2. X-ray diffraction study

**Fig. 3:**
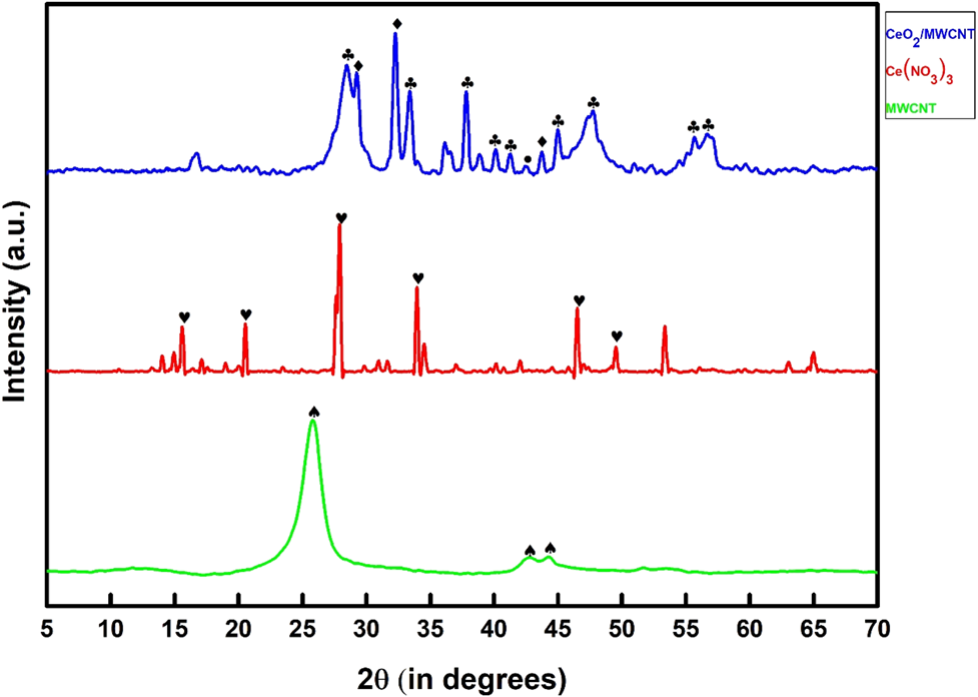
The XRD spectrograph of MWCNT, cerium nitrate, and advanced hybrid CeO_2_/MWCNT nanocomposite powder.

Using standard XRD data files (JCPDS), the identification of distinct phases consisting of functionalized MWCNT, cerium nitrate, and the synthesized advanced hybrid CeO_2_/MWCNT nanocomposite powder was studied. Fig. 3 depicts the X-ray spectrograph of MWCNT, cerium nitrate, and advanced hybrid CeO_2_/MWCNT nanocomposite powder. The characteristic peak of MWCNT is shown at 2*θ* = 42.520 (JCPDS no. 26-1083,•).^33^ The XRD analysis of cerium nitrate shows a single phase with the characteristic peak of cerium nitrate pentahydrate (JCPDS no. 31-0335  ) at 2*θ* = 27.9, 33.96, and 44.5 respectively. The advanced hybrid CeO_2_/MWCNT nanocomposite showed two main phases of cerium oxide CeO_2_ (cerianite syn) having JCPDS no. 43-1002 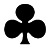 and 32-0196♦ at 2*θ* = 32.14, 24.48, and 37.8 respectively. It also shows the characteristic peak of MWCNT at 2*θ* = 42.520 (JCPDS no. 26-1083, •, ♠).^33^

The developed material showed slight shifting from the original peak of both MWCNT and cerium nitrate, thereby confirming the formation of a new product, *i.e.*, *in situ* development of CeO_2_ nanoparticles. The peaks of the hybrid material ensure the appearance of CeO_2_ nanoparticles (43–1002). Also, when peaks of hybrid materials show a long peak at 2*θ* = 32.8, a variation and shifting from the short peak of cerium nitrate, 2*θ* = 33.91, confirming the conversion of cerium nitrate into cerium oxide.

### 4.3. Morphological analysis

#### 4.3.1. TEM

**Fig. 4:**
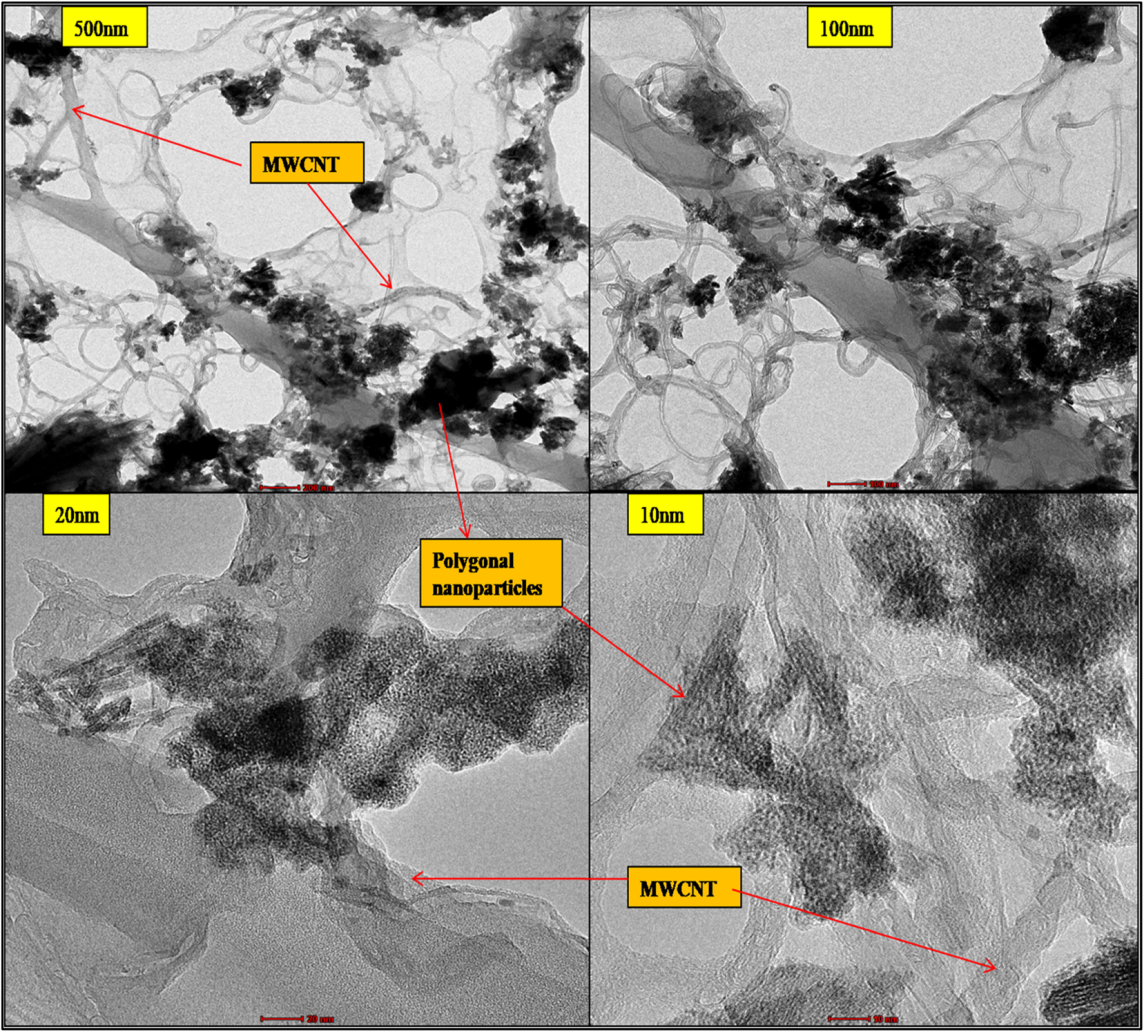
TEM of advanced hybrid CeO_2_-MWCNT nanocomposite.

The advanced hybrid CeO_2_/MWCNT nanocomposite powder was examined using TEM at different *X* magnifications, and the microphotograph is shown in Fig. 4. The microphotographs reveal a unique hybrid organic–inorganic structural morphology possessing nanosized polygonal bunches of hybrid cerium oxide nanoparticles anchoring on the surface attached to carboxyl groups of the MWCNT.^34^ The long curved MWCNTs were densely, securely, and effectively impregnated on the walls of MWCNTs with polygonal morphology cerium oxide nanoparticles.^34^ This novel hybrid material with MWCNT coated with the required high-density nanomaterial, *i.e.* metal-oxide, shows optimal radiation reduction in the generated sample.

The detailed structure is further revealed in microphotographs of 20 and 10 nm; the nano polygonal honeycomb structures are visible to be anchoring on the walls of MWCNT and are hanging down as well, which depicts the successful synthesis of the hybrid nanocomposite. Due to the higher dispersion, some slight agglomeration in the nanoparticles is also seen in the pictures. This can be overcome by varying the ratio of MWCNTs and cerium source taken during the initial fixation of the reaction's chemical composition, and the process parameters can also be revised.

#### 4.3.2. SEM

**Fig. 5:**
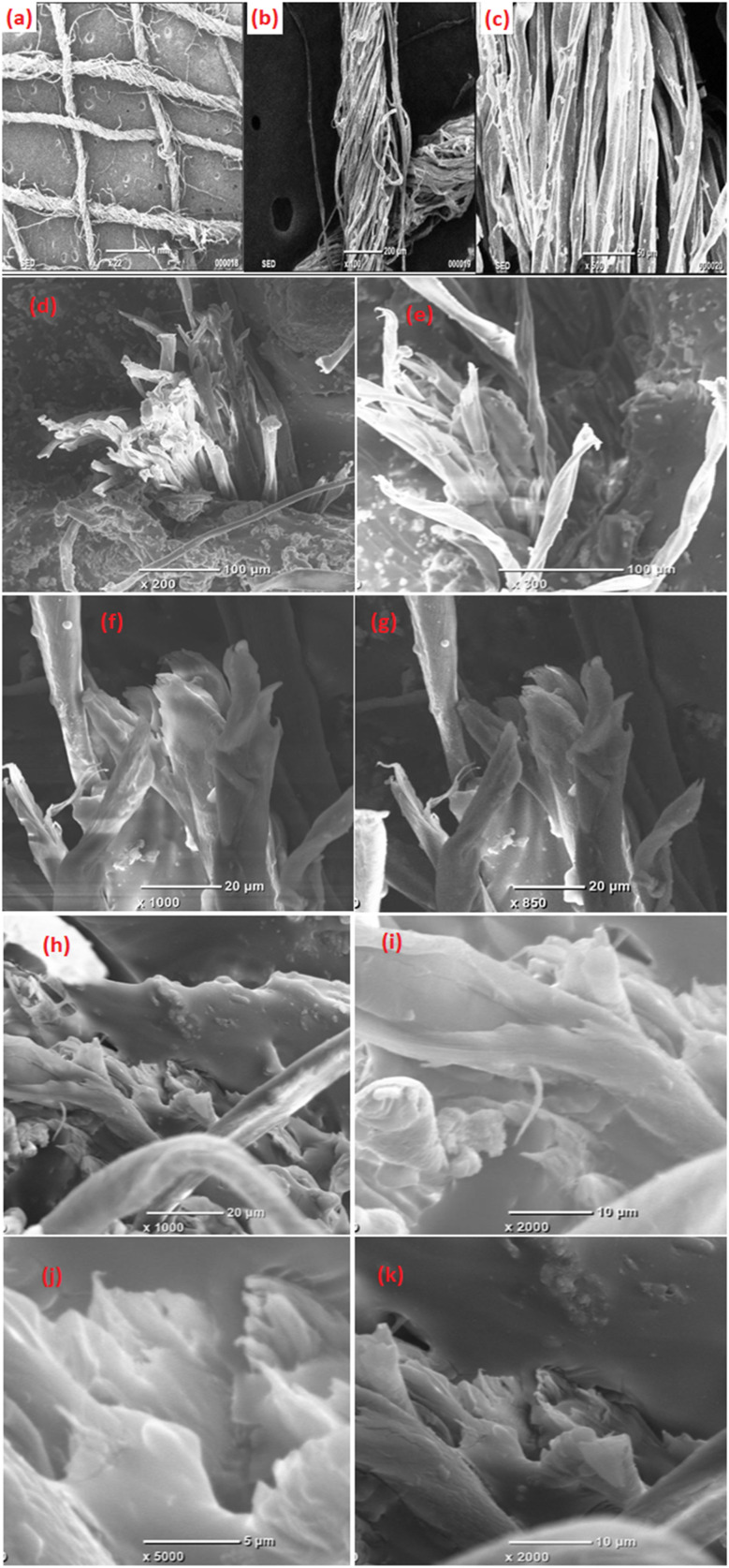
(a–c) SEM images of untreated cotton bandage (washed and sun-dried); (d–g) SEM images of the fractured surface of the conventional bandage with MWCNT (A) at magnification 200, 300, 850, and 1000; (h–k) SEM images of conventional bandage coated with hybrid material, *i.e.*, CeO_2_-MWCNT (B), at magnification 1000, 2000, and 5000.

The surface of a pure cotton bandage, a conventional bandage created with MWCNT/PDMS, and a conventional bandage covered with a hybrid material, *i.e.*, CeO_2_-MWCNT/PDMS, were studied using SEM, and Fig. 5 shows the magnified images. Size distributions of Ce and MWCNT particles and the layered composites with varying layer thickness ratios and layered interfaces are illustrated in Fig. 5. The SEM image of an untreated cotton bandage at magnifications 22×,100, and 500 depicts smooth, ribbon-like threads Fig. 5(a–c). The tiny empty spaces are seen in the untreated fibers and the fibers.^14^ These spaces are then further filled with the developed jelly material and into the fabric to develop the desired sample. As a result, it contributes to the created traditional bandage coated with the required material's flexibility, more mechanical strength, and higher X-ray shielding characteristics. Visualizing SEM microphotograph of the coated bandage, the appropriate infusion of the generated jelly substance on the surface of the cotton garments proved its proper infusion. The SEM of the cracked surface of the bandage coated with MWCNT/PDMS was studied at different magnifications to evaluate the morphology and adhesion qualities of the jelly substance in the pure cotton fiber which is depicted in Fig. 5(d–g). Also, the SEM of the conventional bandage coated with hybrid material, *i.e.*, CeO_2_-MWCNT/PDMS, is shown in Fig. 5(h–k), confirming an excellent gel dispersion on the fibers of the cotton bandage. At greater magnification, Fig. 5(k), it can be determined that the gel produced is adequately marketed to the fiber and builds a persistent adhesion, which is why it transmits improved strength and radiation protection properties to the combined CeO_2_-MWCNT bandage.

#### 4.3.3. EDS and elemental mapping

**Fig. 6:**
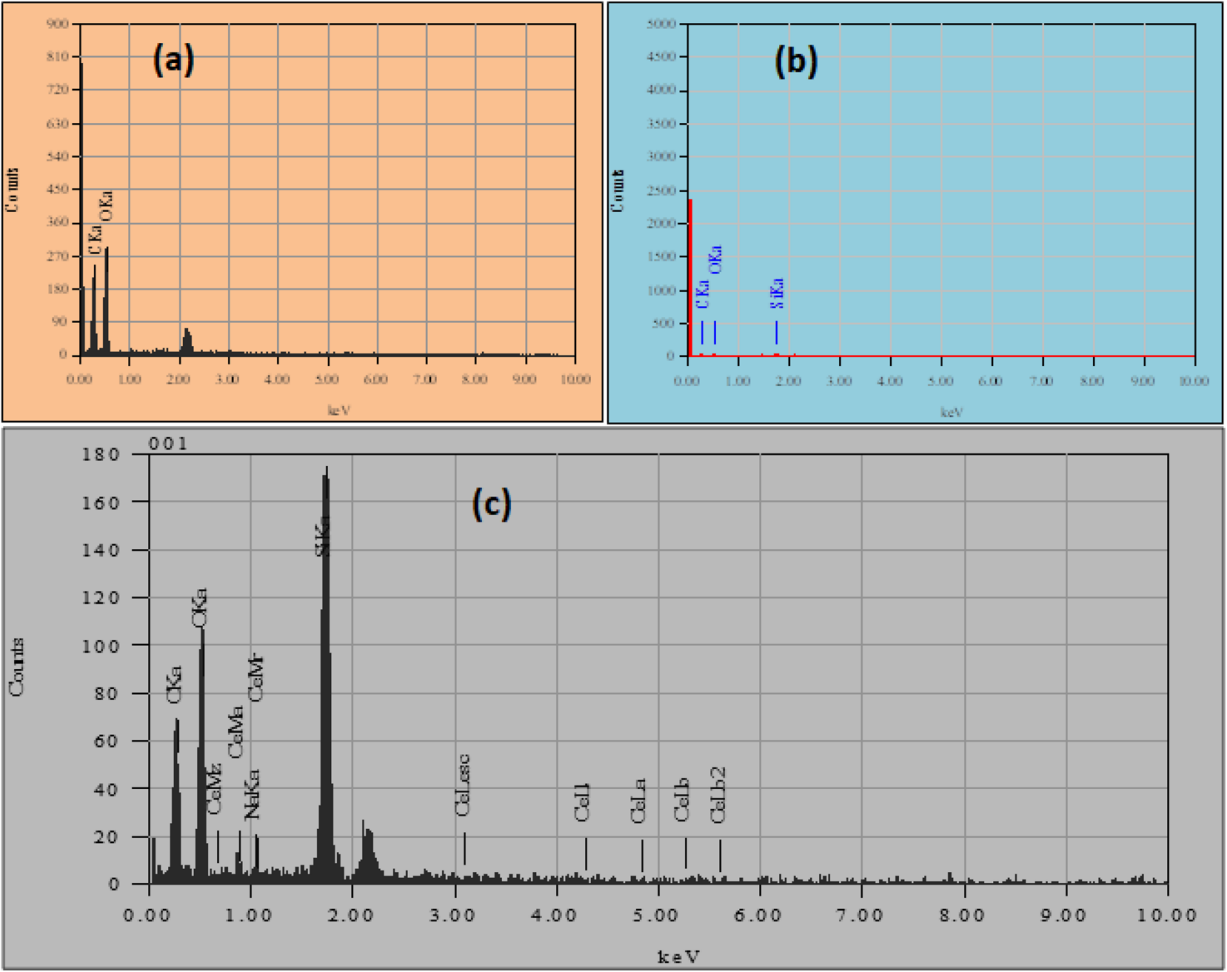
EDX of (a) pure cotton bandage (b) conventional bandage with MWCNT/PDMS and (c) conventional bandage coated with hybrid material, *i.e.*, CeO_2_-MWCNT/PDMS.

The EDS spectra and elemental mapping of the untreated cotton bandage's surface and developed conventional bandage with MWCNT/PDMS and conventional bandage coated with hybrid material, *i.e.*, CeO_2_-MWCNT/PDMS, were examined from a selected area were studied for the quantitative measurements of elements present in it and is depicted in Fig. 6(a–c). The spectra obtained by the EDX data show peaks corresponding to the elemental composition profile consisting of carbon, oxygen, cerium, sodium, and nitrogen and are responsible for making up the composition of the sample being analyzed. Furthermore, the new material's EDX pattern contained carbon and oxygen in the pure cotton bandage.

**Fig. 7:**
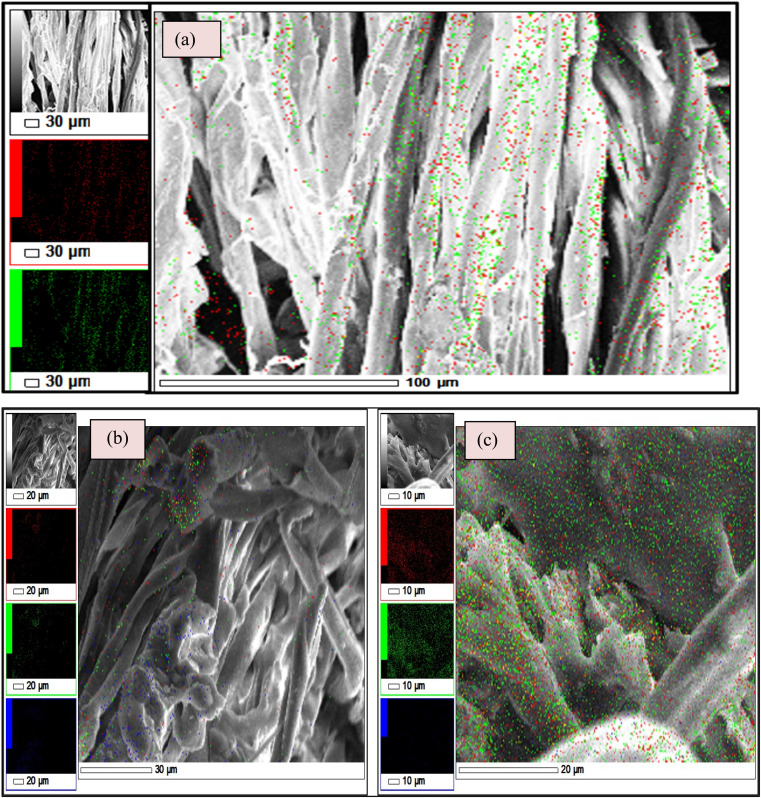
Elemental mapping in the (a) untreated cotton bandage, (b) bandage with MWCNT/PDMS and (c) bandage coated with hybrid material, *i.e.*, CeO_2_-MWCNT/PDMS.

The EDX elemental mapping of both the coated conventional bandage with MWCNT/PDMS and the conventional bandage coated with hybrid material, *i.e.*, CeO_2_-MWCNT/PDMS, was studied and depicted in Fig. 7(a–c). It confirms the distribution of carbon, oxygen particles of MWCNT, and silicon particles from PDMS on the coated cotton bandage surface (A), as in Fig. 7(a). Further, the distribution of carbon, oxygen, and cerium particles on the surface of cotton bandage when coated with hybrid CeO_2_/MWCNT/PDMS, respectively. Elemental mapping confirmed the uniform distribution of all elements (*i.e.*, C, Si, and O on MWCNT/PDMS; and C, O, and Ce on hybrid CeO_2_/MWCNT/PDMS). These images confirmed the *in situ* formation of the expected system, CeO_2_.

Fig. 7 shows that the different colours represent different elements associated with the synthesized material in the elemental mapping. The red colour represents carbon's presence, and the green shows oxygen in the materials. Also, blue colour represents the presence of silica that was contained in PDMS and cerium. It is observed here that the dispersion of cerium on the surface of the synthesized material, thereby making it denser. All the materials (treated/untreated) are mainly made up of cellulose (cotton bandage); thus, they all contain carbon and oxygen.

### 4.4. X-ray shielding and attenuation properties

**Fig. 8:**
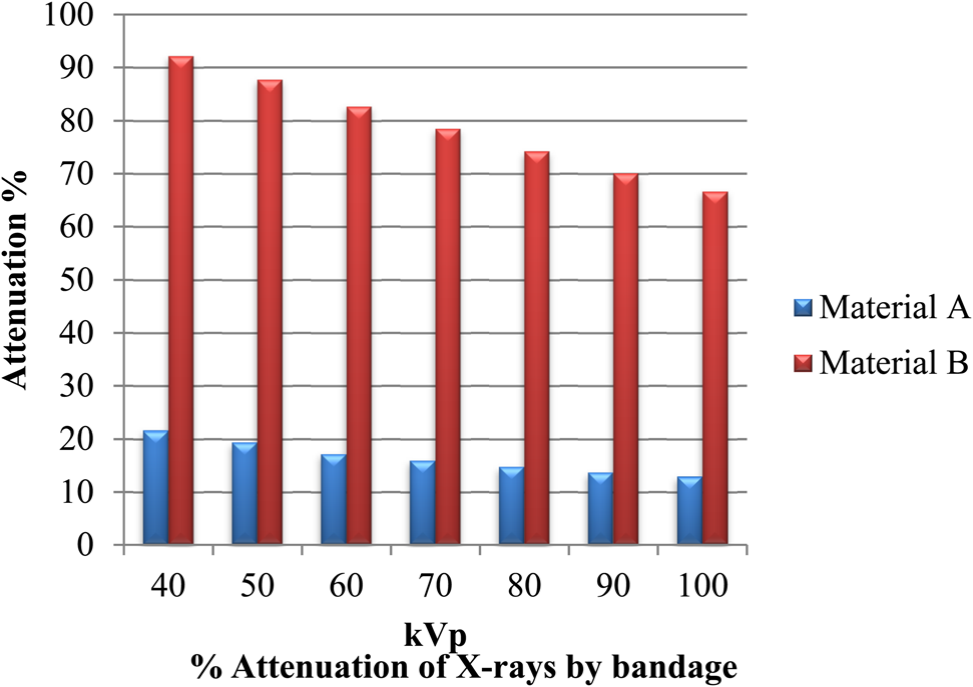
Attenuation % of X-ray by bandage developed *i.e.*, material A conventional bandage coated with MWCNT/PDMS, material B conventional bandage coated with synthesized hybrid material, *i.e.*, CeO_2_/MWCNT/PDMS.

**Fig. 9:**
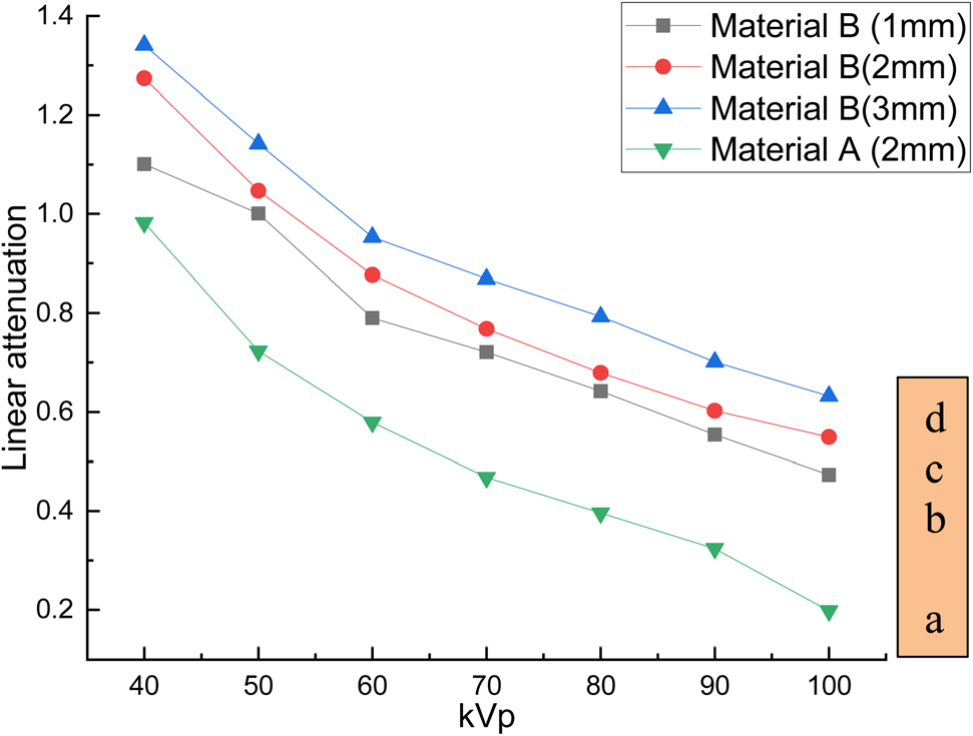
Linear attenuation of (a) developed materials *i.e.*, conventional bandage coated with MWCNT/PDMS, and a conventional bandage covered with synthesized hybrid material, *i.e.*, CeO_2_-MWCNT/PDMS of various thicknesses (b–d) and at different kV.

**Fig. 10:**
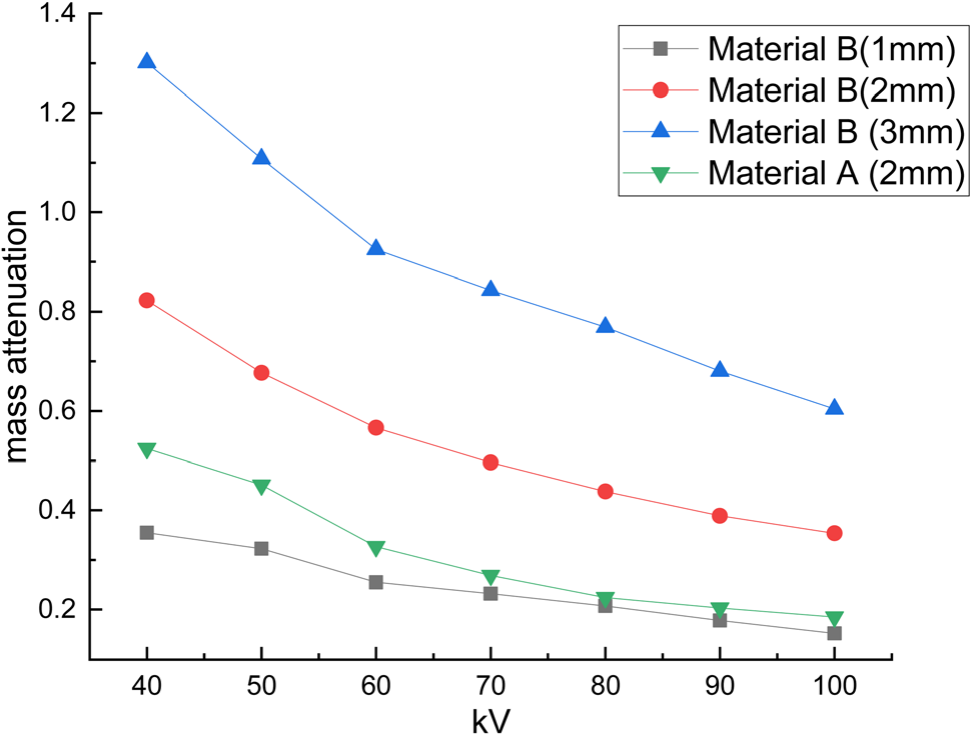
Mass attenuation of (a) developed material *i.e.*, conventional bandage coated with MWCNT/PDMS, and a conventional bandage covered with synthesized hybrid material, *i.e.*, CeO_2_-MWCNT/PDMS of different thicknesses (b–d) and at different kV.

The developed bandages, *i.e.* conventional bandages coated with MWCNT/PDMS and conventional bandages coated with synthesized hybrid material, *i.e.*, CeO_2_/MWCNT/PDMS, were studied for their X-ray radiation attenuation characteristics from 40 to 100 kVp energy. Various parameters like half value thickness (HVL), linear attenuation, and mass attenuation were studied at different kVp (peak voltage) and depicted in Fig. 8–10 below. The attenuation percentage of pure conventional cotton bandage, a conventional bandage coated with MWCNT/PDMS (material A), and a conventional bandage coated with synthesized hybrid material, *i.e.*, CeO_2_-MWCNT/PDMS (material B), was studied as shown in Fig. 8 below.

The result depicts the radiation attenuation characteristics of bandage A, decreasing with the rise in energy. The figure shows that bandage A shows more than 20% attenuation at 40 kvp of X-ray energy. But it falls to 15% with the increase in the energy till 100 kVp the attenuation. On the contrary, improved results are obtained in the developed bandage B, showing an increased attenuation of more than 90% at 40 kvp of X-ray energy. With the rise in the energy to 100 kVp, the attenuation becomes 68%. Thus, both bandages A and B show attenuation characteristics. The bandage B with coated MWCNT/cerium oxide nanoparticles with PDMS flaunted has much more significant attenuation due to the presence of high-density cerium oxide nanoparticles incorporated with MWCNT. It is due to the increased photoelectric absorption and Compton effect. As the photon absorption is directly proportional to the attenuation medium, thus the presence of high-density material results in higher attenuation. The linear and mass attenuation of the developed bandages A and B were also studied, as depicted in Fig. 9 and 10 below. Bandage B was also developed and studied with various thicknesses from 1 mm to 3 mm.

On the other hand, as can be observed, the quantity of attenuation has grown significantly as the layer thickness has increased, particularly in samples containing nanoceria. For samples containing nanoceria, the more significant attenuation is owing to the layers' thickness being significantly more evident. Increased density of nanoparticles in the pathway of radiation may explain this development. Compared with the percentage reduction in magnitude in the samples produced, the critical A-samples may be significantly smaller than the B-sample material of equal thickness.

The linear and mass attenuation percentage linearly decreases with an increase in the kV, as mentioned in Table 1. Also, it is assumed that the rise (alteration) in the thickness of the material leads to an increase (alteration) in the attenuation percentage.^35,36^ From the above figures, it can be verified that with the increase in thickness of the material, *i.e.*, 3 mm, there is an effective increase in the linear and mass attenuation.^37^ It can also be concluded from the values (Table 1) that the attenuation of hybrid material B (conventional bandage covered with synthesized hybrid material, *i.e.*, CeO_2_-MWCNT/PDMS) is comparable to that of Pb composite as reported in the literature. As L. Büermann *et al.* reported determining to lead equivalent values according to IEC 61331-1:2014.^38^ Lead-based composite is highly carcinogenic, hazardous, irritating, and even causes skin diseases. It also produces toxic and dangerous waste products and causes environmental problems. Thus, a hybrid bandage could be effectively utilized as a non-toxic, flexible, and moldable radiation shielding material.

**Table 1:** Linear and mass attenuation of the developed materials, *i.e.*, conventional bandage coated with MWCNT/PDMS, and a conventional bandage covered with synthesized hybrid material, *i.e.*, CeO_2_-MWCNT/PDMS at different kVp (2 mm thickness)

S. no.	kVp	Linear attenuation (in m^−1^)		Mass attenuation (in m^2^ kg^−1^)		Pbbased composite attenuation	References
		MWCNT/PDMS	CeO_2_/MWCNT/PDMS	MWCNT/PDMS	CeO_2_/MWCNT/PDMS		
1	40	0.982	1.274	0.524	0.823	1.386	
2	50	0.723	1.047	0.451	0.6767	1.155	
3	60	0.579	0.877	0.327	0.566	1.019	42
4	70	0.468	0.768	0.269	0.496	0.866	4
5	80	0.396	0.679	0.224	0.438	0.564	

**Fig. 11:**
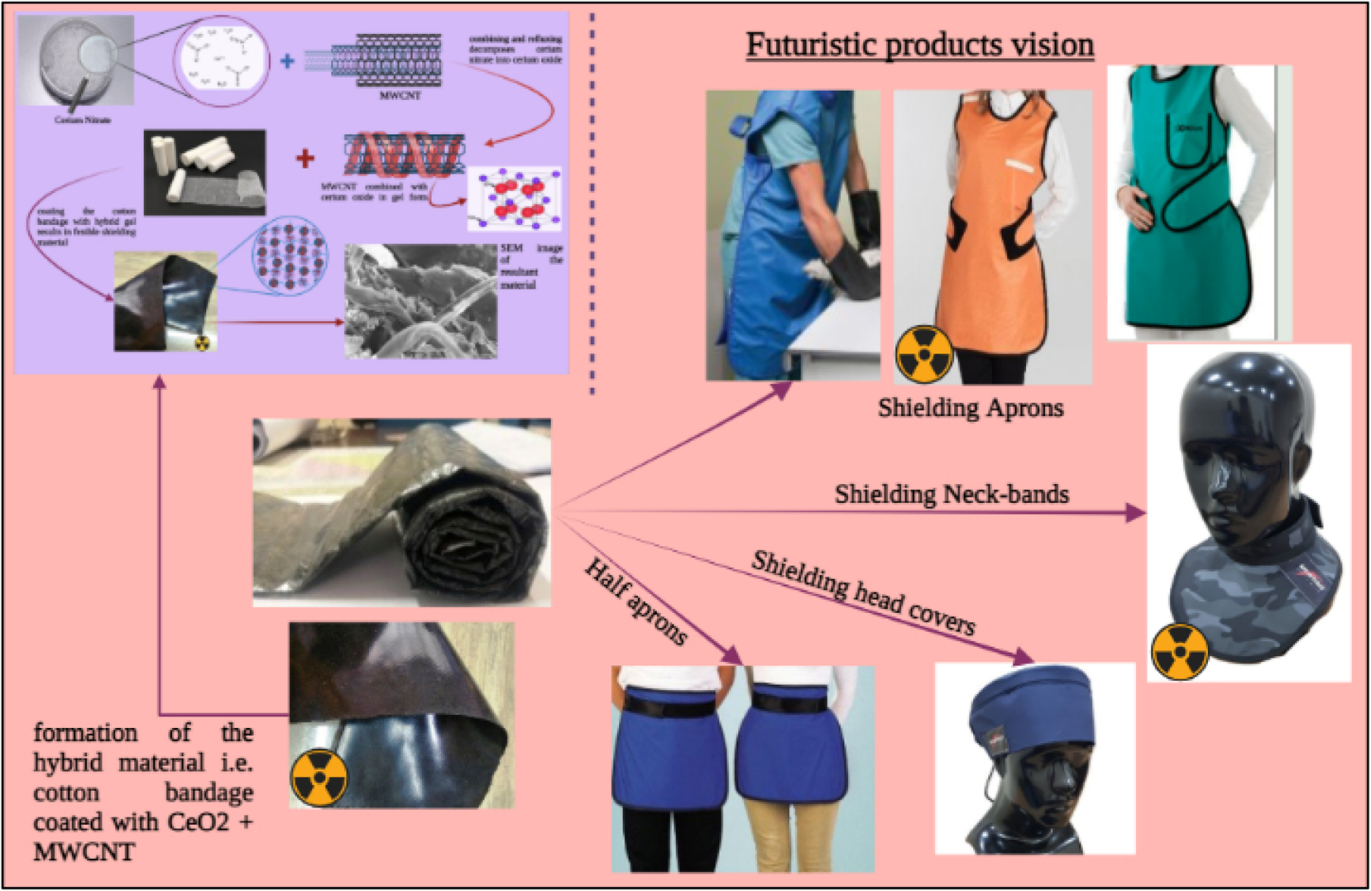
Futuristic products vision using the developed advanced flexible and moldable bandage by harnessing *in situ* synthesized cerium oxide nanoparticles/MWCNTS Nanocomposite for Healthcare Applications.

The performance of the setup utilized to test the samples for X-ray shielding was investigated, as well as the attenuation capabilities of the samples in terms of mass and linear attenuation. Also, the obtained value was compared to the attenuation of the lead composite materials at different kVps, and the attenuation was found to be comparable to that of lead-based products. Thus, this development can easily and efficiently replace lead-based materials. Also, using the developed material, various shielding products (Fig. 11), such as shielding aprons, head covers, *etc.*, can be designed and developed for biomedical sectors.

### 4.5. Possible mechanism

Based on the above results obtained, the possible mechanism for the *in situ* development of cerium oxide impregnated MWCNT coated hybrid material that successfully showed X-ray shielding is as follows:

The initial chemical reaction dissolves cerium nitrate hexahydrate in the water, producing cerium(iii)dihydroxonitrate and nitric acid. The developing cerium hydroxide molecules followed the reaction to further produce the growth of CeO crystallites.^39^ Additionally, after attaining a suitable heating temperature, Ce(OH)_3_ forms CeO_2_ crystallites. In the acidic aqueous solution, cerium ions react readily with water to form cerium hydroxide. Thus, the formed nitrate ions decompose into nitrogen dioxide and oxygen gas during dehydration, as mentioned in the literature.^40,41^

Afterward, the functionalized MWCNT was added to the above acidic aqueous solution and was kept for ultrasonication. The ions present in the solution were:Ce ^3+^(aq.) + 4 NO^3−^ + 6H_2_O + MWCNT

The above solution was mixed with the HMTA (Hexamethylenetetraamine) at room temperature to give a cerium–HMTA complex. Here, the HMTA serves effective additive to provide a controlled morphology of the nanoparticle being produced. The reaction is given by-

5 Ce^3+^(aq.) + 4NO^3−^ + 6H_2_O + MWCNT + C_6_H_12_N_4_ + *x*H_2_O → [Ce(NO_3_)_2_(H_2_O)_5_] (C_6_H_12_N_4_)_2_(NO_3_)(H_2_O)_3_ complex

The decomposition of this cerium–HMTA complex is a multi-step process.^42,43^ The non-coordinated water molecule (3 molecules) in the above complex and the nitrate group gets disintegrated in the first step with a slight increase in temperature (40–150 °C), given by chemical reaction (6) below:

6 [Ce(NO_3_)_2_(H_2_O)_5_]·(C_6_H_12_N_4_)_2_(NO_3_)(H_2_O)_3_ → [Ce(NO_3_)_2_(H_2_O)_5_]·(C_6_H_12_N_4_)_2_

With the increase in the temperature, the complex starts to lose molecules of HMTA, water, and nitrate, resulting in the oxidation of cerium, thus the formation of cerium-oxide, as shown in the reaction below-

7 [Ce(NO_3_)_2_(H_2_O)_5_]·(C_6_H_12_N_4_)_2_ → CeO_2_

The formed cerium oxide was impregnated with the functionalized MWCNT to develop an advanced hybrid CeO_2_/MWCNTs nanocomposite. This gel was then coated on the surface of untreated cotton bandage many-folds to give the resulting X-ray shielding material. Also, it was seen that the OH^−^ concentration affects the formation of unit crystal and their growth, thereby contributing to the material's morphology. Thus, the selection and amount of chemicals (proper ratios) play an essential role in developing desired material.

## 5 Conclusion

This study develops X-ray radiation shielding and morphological studies of advanced flexible bandages by harnessing *in situ* synthesized polygonal cerium oxide nanoparticles/MWCNTS nanocomposite. The lightweight carbon allotropes-based nanostructured bandage, is completely lead-free, excellent and high-efficiency shielding performance, thin and lightweight, excellent durability and flexibility, and good mechanical strength were developed. PDMS, a silicone rubber, has been chosen as the polymer matrix. The impregnated nanoparticles exhibited excellent dispersion with the increased density in the developed bandage. XRD investigations validated the *in situ* synthesis of CeO_2_ nanoparticles impregnated on MWCNT. The existence of polygonal morphology of cerium nanoparticles and its infusion in MWCNT were demonstrated by TEM micrographs. Proper adhesion of advanced gel materials to cotton fabrics has been observed in SEM micrographs. The detailed characteristics confirm the importance of *in situ* development of CeO_2_ nanoparticles impregnated on MWCNT. The impregnated nanoparticles exhibited excellent dispersion with the increased density in the developed bandage and the developed hybrid bandage. Using two narrow and broad beam geometries, the attenuation was tested and shown to have excellent X-ray attenuation properties. As a result, the use of advanced hybrid X-ray protection bandages has a wide range of functions extending from minimizing radiation leaks, transport boxes, and shutting down intensive protective installation in a public place such as X-ray scanning rooms, computed tomography scanner rooms, biomedical testing equipment, military personnel accommodation, nuclear power plants, and other strategic installations.

## Abbreviations

Computed tomography: CT;
Tap water: TW;
R-Ray fluorescence: XRF;
X-Ray diffraction: XRD;
Scanning electron microscope: SEM;
Transmission electron microscope: TEM;
Energy-dispersive atomic X-ray: EDAX;
Peak voltage: kVp

## Data availability

The data that supports the findings of this study are available in the ESI† file.

## Author contributions

S. Verma: conceptualization, data analysis and interpretation, writing – original draft. M. Dhangar, H. Bajpai, K. Chaturvedi: carried out the experiment. R. K. Mohapatra, M. A. Khan: data analysis and interpretation. M. Azam, S. I. Al-Resayes, A. K. Srivastava: writing – review & editing.

## Conflicts of interest

There are no conflicts to declare.

## Supplementary Material

RA-013-D3RA00067B-s001
