# Supplementary material for: X-ray radiation shielding and microscopic studies of flexible and moldable bandage by in situ synthesized cerium oxide nanoparticles/MWCNTS nanocomposite for healthcare applications
Source: RSC Adv. 2023 Mar 15;13(13):8594–605. doi: 10.1039/d3ra00067b (PMC10016082; doi:10.1039/d3ra00067b)
Supplement: RA-013-D3RA00067B-s001 [file RA-013-D3RA00067B-s001.pdf]

Electronic Supplementary Material (ESI) for RSC Advances.
This journal is © The Royal Society of Chemistry 2023

X-ray radiation shielding and microscopic studies of flexible and moldable bandage by in-situ synthesized Cerium oxide nanoparticles /MWCNTS nanocomposite for healthcare applications

Sarika Verma[a,b,*], Manish Dhangar[a], Harsh Bajpai[a], Kamna Chaturvedi[a], Ranjan K. Mohapatra[c,*], Mohd. Akram Khan[a,b], Mohammad Azam[d,*], Saud I. Al-Resayes[d], Avanish Kumar Srivastava[a,b]

[a]*Council of Scientific and Industrial Research- Advanced Materials and Processes Research Institute, Hoshangabad Road, Bhopal (M.P.), 462026, India.*

[b]*AcSIR-Advanced Materials and Processes Research Institute (AMPRI), Hoshangabad Road, Bhopal (M.P) 462026, India*

[c]*Department of Chemistry, Government College of Engineering, Keonjhar-758002, Odisha, India*

[d]*Department of Chemistry, College of Science, King Saud University, PO BOX 2455, Riyadh 11451, Saudi Arabia*

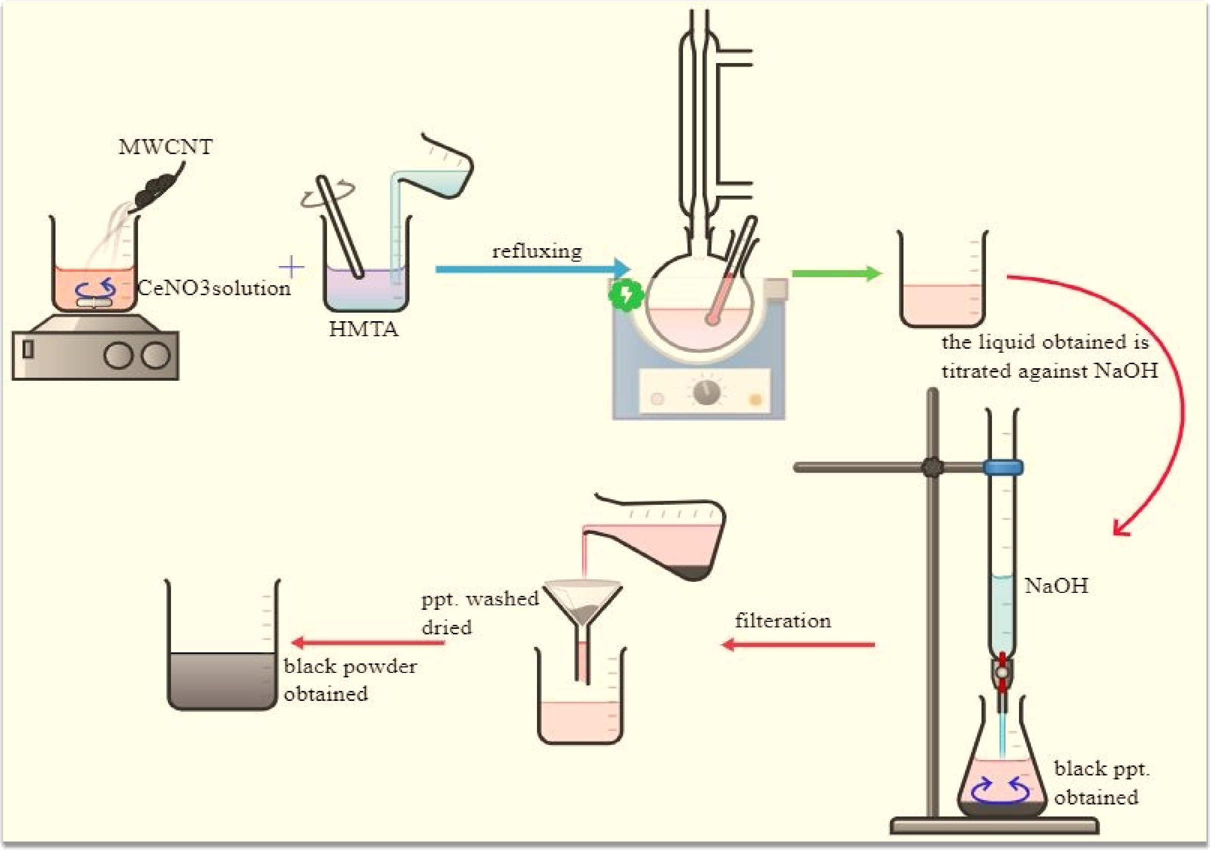

Fig. S1. Preparation of advanced hybrid CeO_2/MWCNTs nanocomposite
